# Advanced supervised machine learning methods for precise diabetes mellitus prediction using feature selection

**DOI:** 10.3389/fmed.2025.1620268

**Published:** 2025-09-10

**Authors:** Gufran Ahmad Ansari, Salliah Shafi, Mohd Dilshad Ansari, Azhar Shadab

**Affiliations:** ^1^College of Computer and Information Sciences, Imam Mohammad Ibn Saud Islamic University (IMSIU), Riyadh, Saudi Arabia; ^2^GNA University, Phagwara, Punjab, India; ^3^SRM University Delhi-NCR, Sonepat, Haryana, India; ^4^G.L. Bajaj Institute of Technology and Management, Greater Noida, India

**Keywords:** cross validation, diabetes, diabetes mellitus, K-Nearest Neighbors, machine learning techniques, Naive Bayes, prediction, supervised

## Abstract

**Background:**

Diabetes mellitus (DM) is a chronic metabolic disorder that poses a significant global health challenge, affecting millions, many of whom remain undiagnosed in the early stages. If left untreated, diabetes can result in severe complications such as blindness, stroke, cancer, joint pain, and kidney failure. Accurate and early prediction is critical for timely intervention. Recent advancements in machine learning techniques (MLT) have shown promising potential in enhancing disease prediction due to their robust pattern recognition and classification capabilities.

**Materials and methods:**

This study presents a comparative analysis of supervised MLT such as Support Vector Machine (SVM), Naïve Bayes (NB), K-Nearest Neighbors (KNN), and Random Forest (RF) using the Pima Indian Diabetes dataset (PIDD) from the UCI repository. A 10-fold cross-validation approach was employed to mitigate class imbalance and ensure generalizability. Performance was evaluated using standard classification metrics: accuracy, precision, recall, and F1-score.

**Results:**

Among the evaluated models, SVM outperformed the others with an accuracy of 91.5%, followed by RF (90%), KNN (89%), and NB (83%). The study highlights the effectiveness of SVM in early diabetes prediction and demonstrates how model performance varies with algorithm selection.

**Conclusion:**

Unlike many prior studies that focus on a single algorithm or overlook validation robustness, this research offers a comprehensive comparison of popular classifiers and emphasizes the value of cross-validation in medical prediction tasks. The proposed framework advances the field by identifying optimal models for real-world diabetes risk assessment.

## 1 Introduction

Diabetes mellitus (DM) is a dangerous chronic disease characterized by elevated blood glucose levels. It can arise due [124mm] Q10to genetic factors or environmental triggers (1). The disease occurs when pancreatic beta cells fail to produce sufficient insulin, impairing the body's ability to regulate glucose levels. Uncontrolled diabetes leads to severe complications, including damage to the kidneys, eyes, blood vessels, nerves, and heart. It also increases the risk of hypertension, foot ulcers, pancreatic disorders, and vision loss (2). Although diabetes affects individuals across all age groups, it has a pronounced impact on older adults. Common symptoms include frequent urination, excessive thirst, and increased hunger (3, 4). If left untreated, the disease can severely impact quality of life. In many developing countries, access to laboratory diagnostic tools such as fasting blood sugar tests and glucose tolerance tests is limited. This lack of early detection infrastructure contributes significantly to diabetes-related mortality (5, 6). Recent years have seen increased application of machine learning techniques (MLT) in disease diagnosis and prediction. MLTs are especially valuable in analyzing medical datasets and identifying patterns that support early diagnosis. They have been effectively applied in predicting diseases such as hepatitis, cancer, and tumors (7). The ability of MLT to derive meaningful insights from structured and unstructured healthcare data enables timely decision-making and personalized care (8).

### 1.1 Types of diabetes

#### 1.1.1 Type 1 diabetes (T1D)

It is a chronic autoimmune condition in which the body's immune system mistakenly attacks and destroys the insulin-producing beta cells in the pancreas, leading to insufficient insulin production. Also known as juvenile diabetes, T1D typically develops in children and young adults under the age of 30, although it can occur at any age. Individuals with T1D require lifelong insulin therapy for survival, as their bodies are unable to regulate blood glucose levels on their own. Key risk factors for T1D include a family history of the disease, pancreatic disorders, and cardiac infections (9).

#### 1.1.2 Type 2 diabetes (T2D)

It is the most prevalent form of diabetes, accounting for over 90% of all diagnosed cases. It occurs when the body becomes resistant to insulin or when insulin is not used effectively, leading to elevated blood glucose levels. T2D is often referred to as insulin-resistant diabetes. According to the National Institute of Diabetes and Digestive and Kidney Diseases, there is a strong association between the rising incidence of T2D and increasing rates of obesity in the global population. Excess body weight and a sedentary lifestyle are among the most common risk factors. The number of individuals affected by T2D will rise significantly worldwide.

#### 1.1.3 Gestational diabetes (GDM)

Diabetes occurs during pregnancy and poses risks to both mother and child. Research indicates that around 18% of pregnant women develop this condition, especially with increasing maternal age (10, 11).

#### 1.1.4 Motivation for study

The International Diabetes Federation (IDF) reported that 700 million people would have diabetes by the year 2045, compared to the current global total of 463 million (9.3% of the total population). Since nearly half of the people with diabetes go undiagnosed, according to the IDF research, 50% of those individuals do not know that they have the disease (International Diabetes Federation). According to estimates, each year, diabetes kills 4.2 million people between the ages of 20 and 79 (38). According to predictions by the International Diabetes Federation (2017), India will have the highest number of people with diabetes (134.3–165.2 million) in 2045. Therefore, there is a critical need to investigate MLT for early diabetes mellitus prediction to support healthcare professionals in providing better diagnoses and treatments for patients.

### 1.2 Why predictive analytics

In terms of database analysis, predictive analytics is the practice of making future predictions based on historical and current data, soft computing, and MLT. It includes directions on how to retrieve intelligence from huge data sets. Because it is all about learning from experience (data), predicting behavior, and offering solutions to various healthcare problems, it has become quite popular. By leveraging vast amounts of information from various data sources and implementing a framework based on an machine learning (ML) framework, it may also be used to increase diabetes prediction accuracy.

### 1.3 Machine learning

A subset of AI, known as machine learning (ML), is used to analyze datasets to generate predictions or take actions to improve certain systems. The primary objective of ML is to train and test the model, then learn from the dataset without explicit input. Based on the learning method, some of the types include supervised learning, semi-supervised learning, exploration of unsupervised learning, and reinforcement learning.

a) Supervised learning: making use of labeled data sets with the desired output, the models (e.g., classifications and regression) are trained and tested.
b) Unsupervised learning: unlabeled data sets without the anticipated consequence are used to train and test the algorithms—for instance, neural networks and clustering.
c) Semi-supervised learning: some models are capable of picking up knowledge from incorrectly labeled data sets. These methods are used for developing models to lower the cost of labeling the data sets.
d) Reinforcement learning: algorithms that use the environment, action, and state to learn through trial and error.

### 1.4 Research context and gap

Despite numerous studies applying MLT for diabetes prediction, many prior studies have notable limitations, including the use of a limited set of performance metrics, a lack of proper validation techniques such as cross-validation (reducing generalizability), inadequate preprocessing and feature selection (leading to reduced model efficiency), and a focus on individual algorithms without comparative analysis.

This study addresses these limitations by:

- Employing four supervised MLT [Support Vector Machine (SVM), K-Nearest Neighbors (KNN), Naïve Bayes (NB), and Random Forest (RF)] for comparison.
- Using 10-fold cross-validation to improve reliability.
- Applying exploratory data analysis (EDA) and preprocessing, including normalization and feature selection.
- Implementing the entire pipeline using the Anaconda (2022) platform and Jupyter Notebook on a Windows OS.

### 1.5 Study contribution

This research proposes a conceptual framework for the early prediction of diabetes mellitus using advanced MLTs. Unlike earlier studies, it emphasizes robust preprocessing, comparative classifier analysis, and practical implementation strategies. The ultimate goal is to improve prediction accuracy and facilitate better decision-making in resource-limited healthcare settings.

The remainder of the study is organized as follows: Section 2 describes the related work on diabetes prediction. Section 3 discusses the methodology for the conceptual framework of MLT. Section 4 describes the results and analysis of different MLTs. Section 5 provides the summary and conclusion, and finally, in Section 6, we outline future research to wrap up the study.

### 1.6 Novelty

Novelty is highlighted in the manuscript. This research introduces a comprehensive conceptual framework for the early prediction of diabetes mellitus using advanced supervised machine learning techniques (MLTs), applied to the Pima Indian Diabetes Dataset (PIDD). Unlike prior studies that primarily focused on single-model performance or basic classification, this study distinguishes itself through several key methodological advancements.

Robust data preprocessing: the framework applies systematic data cleaning, normalization, and handling of missing values, ensuring the dataset is optimized for learning algorithms and minimizing bias introduced by noisy data.

Comparative classifier analysis: a detailed evaluation of multiple models—SVM, RF, KNN, and NB—was conducted using not only standard performance metrics (accuracy, precision, recall, and F1-score) but also statistical significance testing through McNemar's test. This adds a layer of rigor that is often missing in previous studies.

Feature selection: multivariate analysis techniques are used to identify the most relevant attributes, reducing dimensionality while preserving prediction power, which enhances model interpretability and performance.

Hyperparameter tuning: grid search and cross-validation are employed to fine-tune model parameters, especially for SVM (e.g., kernel type, C value), leading the SVM to achieve 91.5% accuracy.

Practical implementation focus: the framework is designed with scalability in mind, particularly suited for resource-constrained healthcare environments where automated, accurate early diagnosis can substantially improve patient outcomes.

Generalizability: the study outlines a clear roadmap for extending the framework to unstructured data and other diseases, such as cardiovascular conditions, tumors, Parkinson's disease, and COVID-19.

## 2 Related studies

Many scholars around the world have used various approaches to improve diabetes prediction using MLT. Some of the most common ways are as follows: a diabetic condition caused by a spike in blood sugar levels has been explored by Veena Vijay, V., and Anjali C. Using decision trees (DT), SVM, Naive Bayes (NB), and ANN algorithms, various information systems for predicting and detecting diabetes are described (12). P. Suresh Kumar and V. Uma Tejaswi have developed methods for identifying diabetes using MLT, such as SVM, decision trees, and Naive Bayes (13). The HEABCAKSVM (hybrid improved artificial bee colony advanced kernel support vector machine) has been utilized to construct a hybrid predictive model for better diabetes diagnosis (14).

The model's accuracy is 90.04%, and it was constructed utilizing machine learning techniques to develop a hybrid model for T2DM prediction. Jakka and Rani (37) proposed an ingenious solution. The system is designed to forecast T2DM (15). They compare the performance of six MLTs using a variety of metrics. Compared to other classifiers included in the model, Logistic Regression (LR) has the highest accuracy (77.6%). Tigga and Garg (39) used the questions to create diabetic predictive models from the Indian healthcare dataset. To predict diabetes, ML techniques, including LR, K-nearest neighbor (KNN), SVM, Decision Tree (DT), Naive Bayes Classifier, and Random Forest (RF), were applied to data collected by questionnaires and the Pima dataset (16).

To improve the model's ability to explain and classify diabetes risk, it is necessary to adjust the MLT settings. Research on MLT approaches, such as Support Vector Machine, NB, and DT for special disease prediction, has utilized Principal Component Analysis. Nawaz Mohamudally and Dost Muhammad predicted diabetes using the C4.5 decision tree algorithm, a multi-layer perceptron, the K-means clustering technique, and visualization methods (17).

According to a related study, each of these researchers investigated certain techniques and worked to develop methods to the best of their abilities. Using multiple feature selection methodologies, the authors hope to construct a model that can effectively categorize and predict diabetes data at an early stage. Practitioners and researchers employed a variety of ML algorithms to analyze medical data and calculate healthcare costs. To analyze diabetic data, a variety of datasets, including SVM integrated learning models and the DT technique, have been applied. Data were initially collected from individuals who had participated in studies utilizing the SMOTE algorithm, recognized as one of the most effective feature selection methods, alongside an unbalanced approach that considered factors such as age, body mass index (BMI), and blood glucose levels, while also incorporating other important characteristics like smoking habits. Processed characteristics are subsequently evaluated through the previously mentioned data mining classification, which differentiates between normal and abnormal features while maintaining error-free results.

With an Receiver operating characteristic (ROC) value of 89% and a 78.65% accuracy rate, the SMOTE-based diabetes detection method is more accurate and precise than traditional SVM classifiers (18). Most current diagnostic algorithms are built to have a knowledge base gathering a data set, and this has alarmed some academics, including the signs and symptoms of a certain illness. The performance of the prediction system is significantly impacted by the accuracy of the data source. To solve this problem, a basic set-based prediction system was created and implemented. The suggested method uses 19 people's symptoms as input to identify the type of diabetes each one suffers from. The results show that arranging prediction models has been demonstrated to be significantly better than existing rule-based prediction models.

To increase the output of challenging optimization problems, the learning technique variables must be modified. It was also observed that the optimization approach is gaining popularity as a solution for resolving complex problems that are challenging to handle using traditional methods. The performance effectiveness of machine learning techniques has to be developed in two stages. Using a correlation-based feature selection method, it is essential to determine the features that are most relevant in the first stage (19). In the next stage, hyperglycemia and heart disease are classified using the RF method. After a results analysis, it was found that the suggested RF technique significantly increases the accuracy of heart disease and diabetes prediction (20–22). It was also discussed how essential it is to diagnose diabetes early on and the many negative effects of the disease. Early detection of diabetes could potentially assist individuals in reducing their risk of subsequently developing additional conditions, including heart disease, neuropathy, or retinopathy. The difficulties of developing diabetes were examined, along with the value and need of using advanced technologies to predict how the disease may develop (23, 24). The LDA method is used to select more relevant variables that are directly connected to the diabetes status to improve the precision of diabetic prediction.

Furthermore, it has been indicated that the suggested method might be used by doctors as a practical tool to increase their earnings and draw accurate conclusions (25–28). Six MLTs were utilized to analyze the data set and formulate hypotheses regarding predictive analytics in healthcare. An evaluation was conducted to predict diabetes, comparing various ML models (29–32). The performance of SVM and KNN on the PIDD data set demonstrated high accuracy.

The models for hyperparameter tuning aimed at achieving high precision were not considered in this study. In this proposed work, the implementation of four MLTs, viz., SVM, KNN, NB, and RF, was performed, and the analytical outcomes were evaluated in relation to statistical analyses. The implementation and analysis revealed that SVM has the highest accuracy across all classifiers of 91.5%. In Table 1, a comparison of earlier research on performance parameters is shown. The comparative analysis dataset highlights the performance of various machine learning algorithms applied to diabetes prediction using different datasets. The proposed study aims to build upon these results by incorporating optimized feature selection, hyperparameter tuning, and ensemble learning techniques to improve the accuracy and reliability of diabetes prediction models. By comparing traditional and advanced supervised learning approaches, this research seeks to identify the most effective strategies for early and accurate diabetes detection (33).

**Table 1:** Comparison of related studies.

**S. No**.	**Ref No**	**Algorithm used**	**Data set**	**Results**
1.	(20)	RF, MLP, SVM, GB, DT	Clinical dataset	RF80%, MLP89%, SVM87%, GB88%, DT79%
2.	(21)	SVM, DT	PIMA	SVM83%, DT72%
3.	(22)	RF, XGB, DT	PIMA	RF85.5%, XGB90.5%, DT88%
4.	(23)	MLP, Radial basis function	PIMA	MLP 78.1%
5.	(24)	ID3, DT	PIMA	ID3 80.8

Key observations from the previous research of other researchers:

Algorithm comparison: Various studies have implemented classifiers such as Naive Bayes (NB), Random Forest (RF), Decision Trees (DT), Gradient Boosting (GB), Support Vector Machine (SVM), and Multi-Layer Perceptron (MLP) to classify diabetic and non-diabetic patients.

Dataset variability: Most studies have utilized the PIMA dataset, while some have employed clinical datasets for model training and validation.

Performance benchmarking: RF models generally perform well, achieving accuracies between 76 and 85.5%. SVM-based models yield an accuracy range of 83%−87%. XGBoost (XGB) has shown superior performance, reaching 90.5% accuracy in one study.

**Figure 1:**
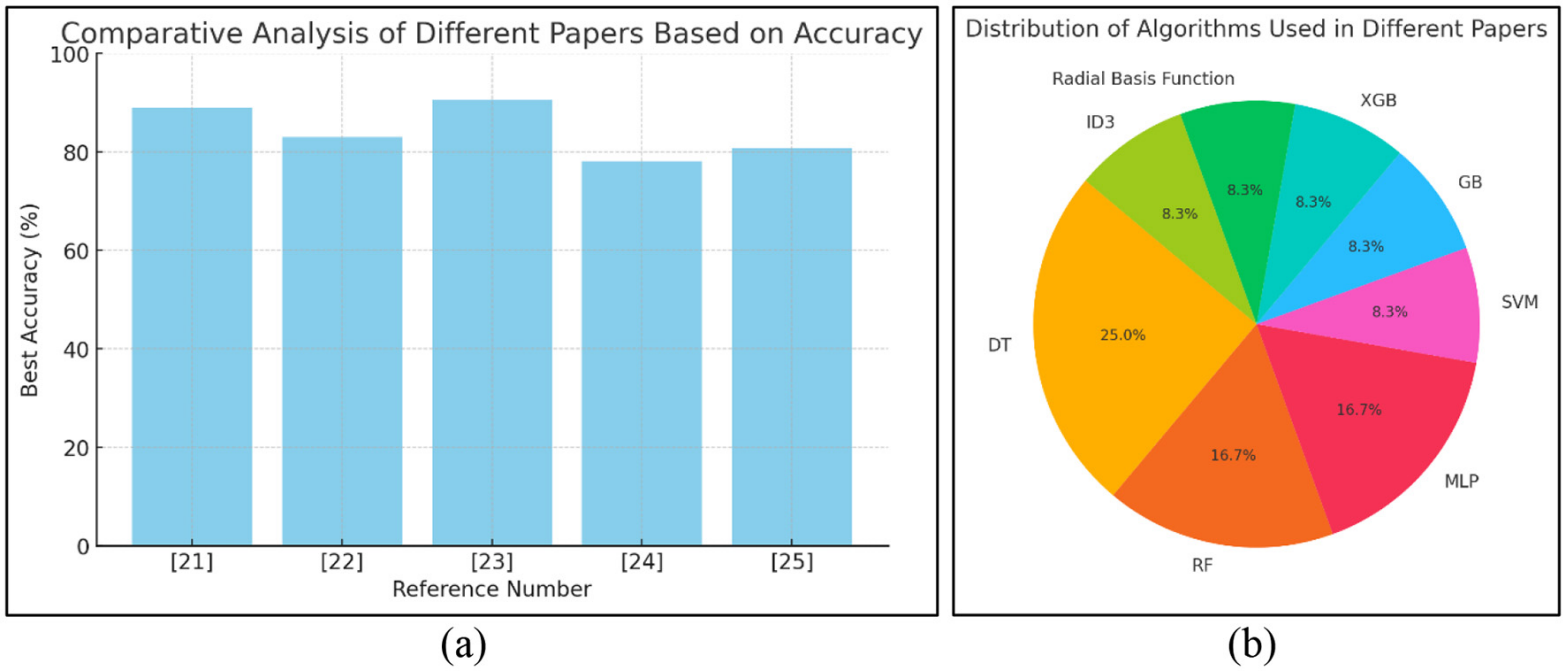
**(a)** Bar chart and **(b)** pie chart comparing the accuracy of different algorithms used in various research papers.

Figures 1a, b show a graphical representation of previous work. The bar chart and pie chart show the use of MLT in diabetes prediction research. A bar chart compares the accuracy of different algorithms across various research papers, providing a clear visualization of their performance. Meanwhile, the pie chart represents the distribution of these algorithms, which highlights their popularity in the referenced studies.

## 3 Methodology for the conceptual framework of MLT

**Figure 2:**
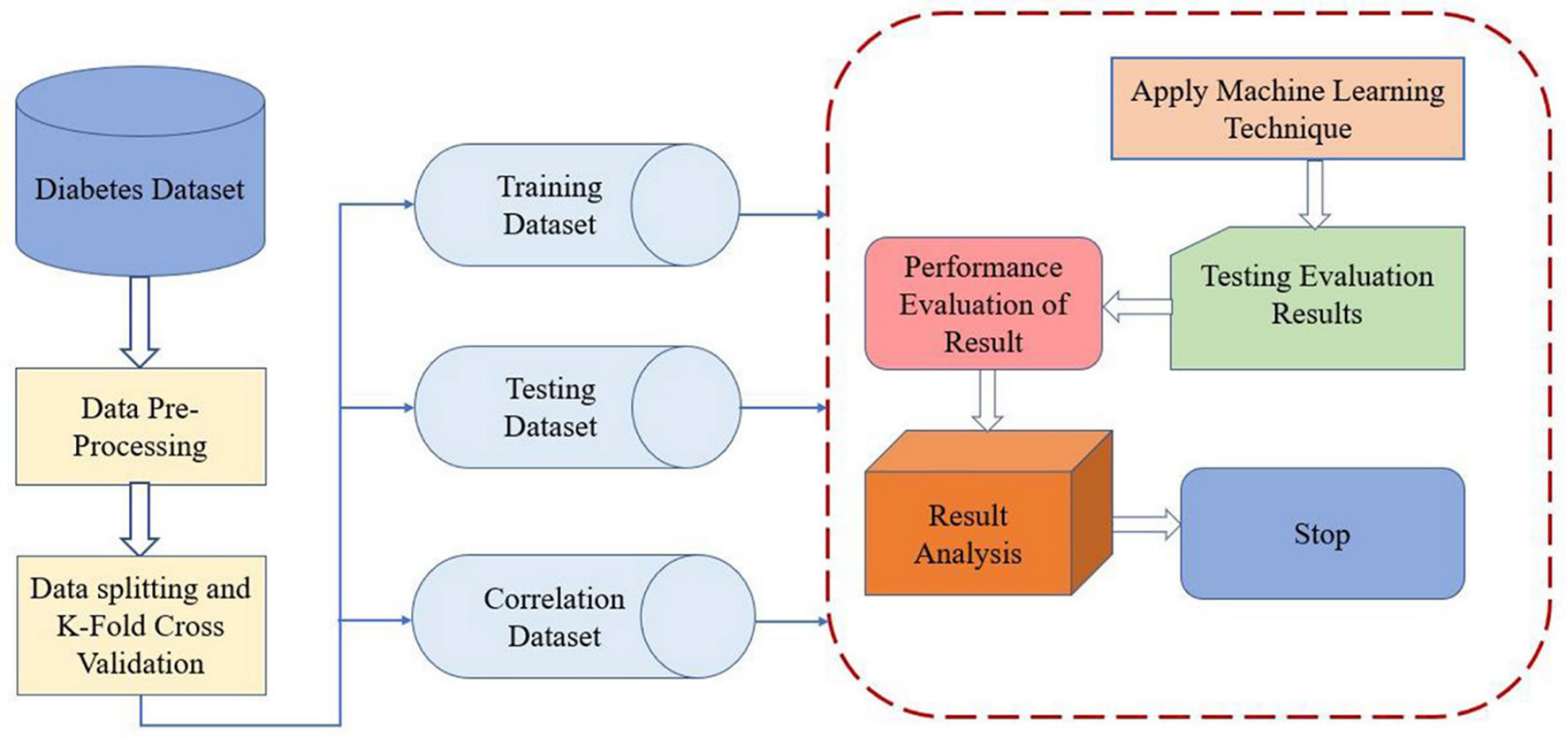
Conceptual framework for MLT.

The framework presented in Figure 2 demonstrates novelty through its thoughtful integration of key machine learning steps tailored for medical diagnosis. It introduces a unique way to split data, separating training, testing, and correlation datasets, allowing for targeted feature analysis before modeling. Incorporating k-fold cross-validation early in the pipeline ensures consistent model validation and reduces overfitting risks. Unlike traditional workflows, the framework emphasizes a layered evaluation approach, distinguishing between performance scoring and result analysis. This promotes better interpretability and reliability in clinical settings. Additionally, its modular structure supports easy adaptation to other healthcare applications.

### 3.1 Dataset and data collection

**Figure 3:**
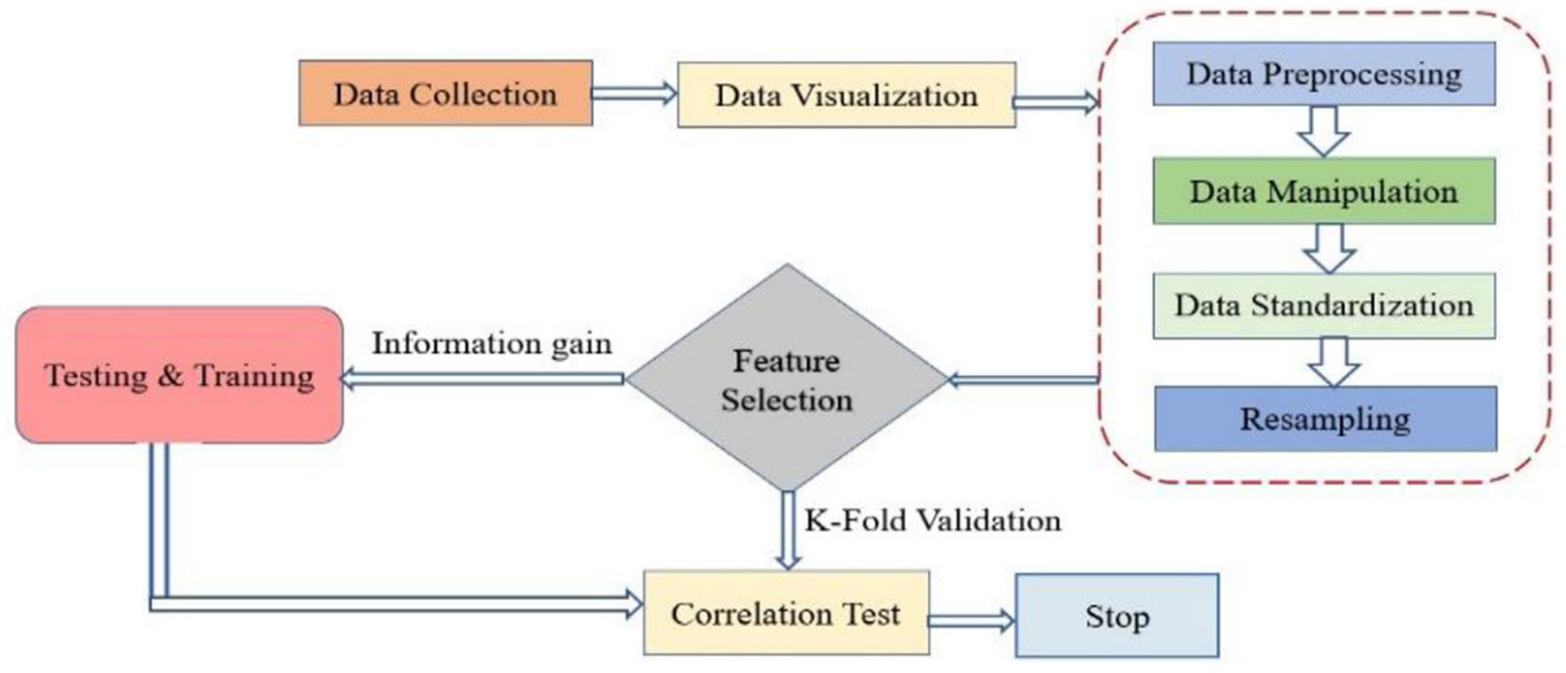
Flow chart of the complete process.

Regarding the observation in Table 2, where all feature values appear as integers, this is indeed accurate for some features, such as pregnancies, age, and glucose, which are naturally recorded in whole numbers. However, features like body mass index (BMI ) and Diabetes Pedigree Function are originally float values (i.e., decimals) in the PIDD dataset. The reason they may appear as integers in Table 2 could be due to either a formatting issue during data presentation or rounding for simplicity in tabular visualization. It is important to note that during actual processing and analysis in the Python environment, data retain their original formats as provided in the CSV file, which include both integer and floating-point data types. The dataset used consists of 419 rows and eight columns, as outlined in Table 2. These parameters include pregnancies, glucose, blood pressure, skin thickness, insulin, BMI, diabetes pedigree function, and age. Data were sourced in Excel format and processed using Python's Pandas library to read a CSV file. Figure 3 illustrates the data processing flow, outlining stages of data import, preprocessing, model training, and evaluation.

**Table 2:** Dataset description for analysis.

**S. No**	**Parameters**	**Description**	**Values**
1	Pregnancies	Number of times pregnant	1, 2, 3….
2	Glucose	The glucose level of a person	1, 2, 3….
3	Blood pressure	Blood pressure status	1, 2, 3….
4	Skin thickness	Skinfold thickness of triceps (mm)	1, 2, 3….
5	Insulin	Whether insulin is needed or not	1, 2, 3….
6	BMI	Body mass index of a person	1, 2, 3….
7	Diabetes pedigree function	Family history of diabetes (a risk factor for diabetes)	1, 2, 3….
8	Age	Age is one of the most essential components of health care	1, 2, 3….

### 3.2 Strategies for accurate prediction of diabetes mellitus

These features are essential for training supervised learning models, enabling the development of an accurate and robust diabetes prediction system. The study likely evaluates different MLT optimizing feature selection and model performance to improve diagnostic reliability.

#### 3.2.1 Data visualization

**Figure 4:**
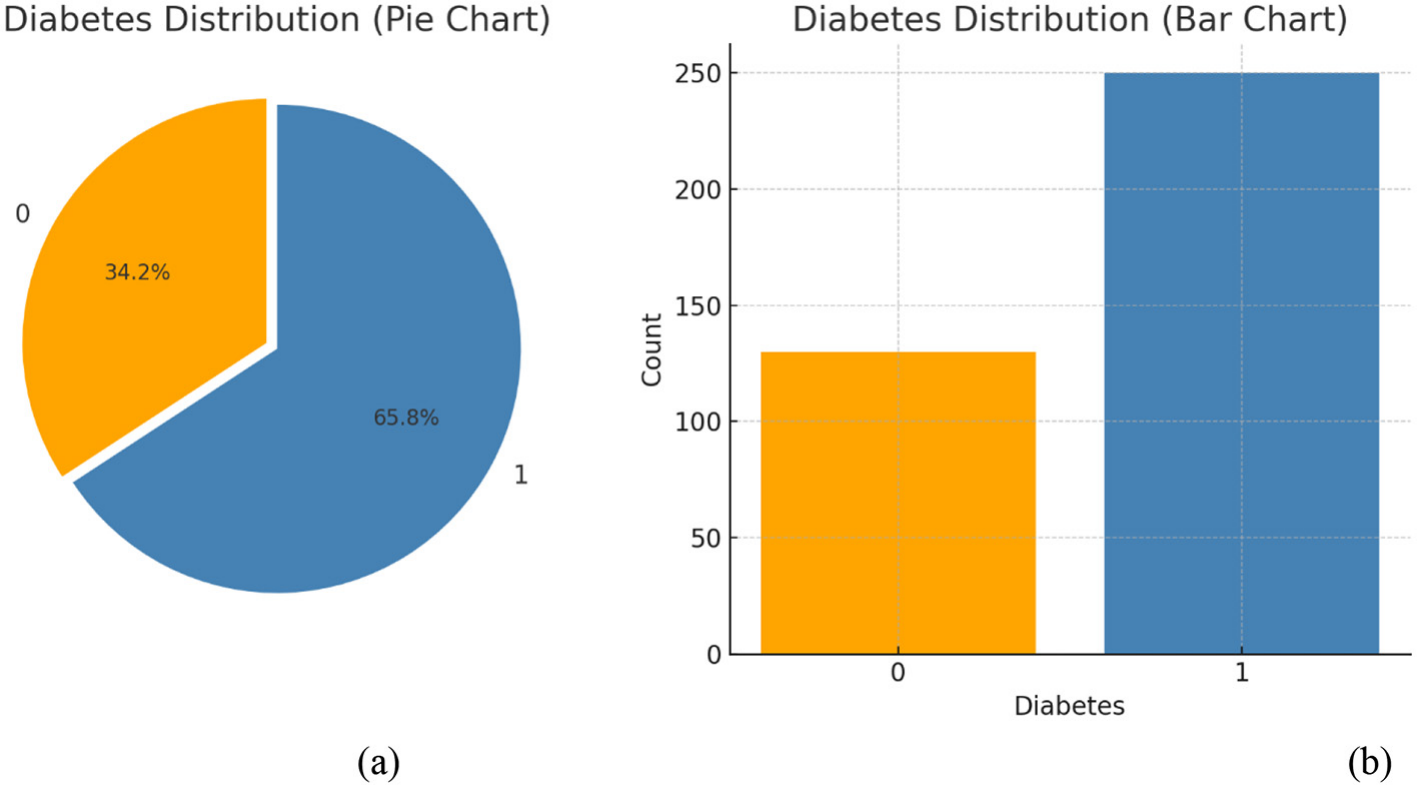
**(a)** Percentages and **(b)** count of diabetes outcomes.

Data visualization enhances awareness by presenting information in an easily understandable visual format. Figure 4 illustrates how the dataset is represented during this phase. The analysis shows the percentage of individuals affected by diabetes and also forecasts the number of future cases. The dataset is stored in a CSV file, which is imported into the notebook using the Python Pandas module. Once data is loaded, various analytical tasks can be performed. To generate visualizations, we use Pandas in combination with graph-plotting libraries. Figure 4 explicitly displays the distribution of the target variable, diabetes, distinguishing between non-diabetic (0) and diabetic (1) cases. The steps for data visualization and standardization are introduced at a basic level.

#### 3.2.2 Data preprocessing

Data preprocessing is a critical step in any machine learning pipeline, as it directly affects model performance and generalizability. In this study, multiple preprocessing techniques were carefully employed to enhance data quality and model effectiveness.

A common concern with the Pima Indian Diabetes Dataset (PIDD) is its age and potential lack of relevance. While it is a well-known benchmark dataset, we acknowledge that it may not fully reflect current population diversity or clinical standards. However, its widespread use allows for reproducibility and valid baseline comparisons. Future studies will involve validating our framework on more recent and real-world datasets to enhance its applicability.

The class imbalance in PIDD, where diabetic cases (positive class) are fewer than non-diabetic cases, was indeed considered. While this imbalance can bias model performance, especially toward the majority class, we addressed this issue using 10-fold cross-validation. This technique partitions the dataset into balanced folds, ensuring that each model evaluation accounts for representative samples of both classes. Additionally, model metrics such as precision, recall, and F1-score were used alongside accuracy to provide a more reliable performance assessment.

A common source of confusion is the simultaneous use of normalization and standardization. These techniques serve different purposes, and in our pipeline, they were applied selectively based on algorithmic needs:

- Normalization was applied during exploratory phases, particularly for distance-based algorithms such as KNN, where scaling all features to a [0, 1] range improves distance calculations and convergence behavior.
- Standardization, on the other hand, was used during final model training, especially for algorithms like SVM and logistic regression, which assume normally distributed input features. This transformation, defined as

$${\text{x}}^{*}\text{= }\frac{\text{x-μ }}{\sigma }\;,$$

where x^*^ is the standardized value, x is the original feature, μ is the feature mean, and σ is the standard deviation, ensures that all features contribute equally to the learning process. Exploratory Data Analysis (EDA) was also conducted to detect outliers, assess feature distributions, and identify correlated variables, all of which informed our feature selection and preprocessing strategies. These steps collectively improve model convergence, accuracy, and generalization.

#### 3.2.3 Data manipulation

Data manipulation involves transforming raw data into a structured format suitable for analysis. In the context of this study, it includes cleaning missing values, removing duplicates, correcting data types, and integrating multiple features where necessary. These steps ensure that the dataset is free from inconsistencies and prepared for further preprocessing stages, such as normalization and model training. Additionally, exploratory data analysis (EDA) was performed to detect outliers, class imbalances, and feature correlations, allowing for informed decisions during feature selection and transformation.

#### 3.2.4 Data standardization

Standardization is a crucial preprocessing step in machine learning that involves rescaling features to have a mean of 0 and a standard deviation of 1, as shown in the following equation:


$$\begin{array}{l} x^{*}=\frac{x-K}{\sigma }, \end{array}$$

where:

- x* is the standardized value
- *k* is the mean of the training data
- σ sigma is the standard deviation of the training data.

This transformation ensures that each feature contributes equally to the model's learning process, which is especially important for algorithms like SVM and KNN that are sensitive to the scale of input variables. Standardization improves convergence and model accuracy while maintaining the relationships between features.

#### 3.2.5 Resampling

Resampling refers to a set of methods used to restore our sample datasets, which include training and validation sets. In this research, RF used to improve accuracy in the PIDD set has achieved the highest accuracy of 91%.

#### 3.2.6 Feature selection

One of the key selection features of the suggested method is the feature selection process. The process of feature selection involves reducing the dimension of the data by selecting suitable features from the raw data feature set based on certain assessment criteria and removing redundancy from the feature set to reduce the dataset dimension. Diabetes occurs due to an excess of insulin or sugar in our blood. Glucose levels remain high whenever the blood is unable to convert sufficient insulin. Glucose aids in cellular digestion, significantly reducing blood glucose levels and providing energy. This research seeks to develop an MLT using data from the Kaggle dataset.

#### 3.2.7 Feature selection using information gain

Feature selection plays a crucial role in improving MLT performance, especially when dealing with high-dimensional medical datasets. The objective is to select the most informative features from the original dataset while eliminating redundant or irrelevant attributes, thereby reducing computational complexity and enhancing prediction accuracy. In this study, information gain (IG) is employed as the primary criterion for feature selection. IG measures the reduction in entropy (or impurity) achieved by splitting the dataset based on a particular feature. Features that contribute to the highest reduction in entropy are considered the most informative and are retained for model training.

Entropy and information gain: entropy quantifies the impurity or disorder in a dataset and is calculated using the following equation:


$$\begin{array}{l} \text{E}\left(\text{T}\right)=\sum_{i=1}^{T}Pi\;\log\;2pi, \end{array}$$

where

- E(T)E(T) is the entropy of the dataset T
- pi is the probability of class i in the dataset.

When data are completely pure (i.e., contain only one class), entropy is zero. Conversely, higher entropy values indicate more impurity or uncertainty.

Information Gain (IG) is then computed by comparing the entropy of the dataset before and after it is split on a particular feature. The steps involved are

- Calculate the initial entropy of the dataset before any splits.
- Split the dataset on each attribute and compute the entropy of each resulting subset.
- Calculate the weighted sum of these entropies.
- Subtract this sum from the original entropy to determine the information gain for that attribute.

$$\begin{array}{l} \text{IG}\left(\text{T},\text{A}\right)=\text{E}\left(\text{T}\right)-\sum \text{values}\left(\frac{Tv}{T}\right)E\;Tv, \end{array}$$

where

- T is the entire dataset,
- A is the attribute being evaluated,
- Tv_ is the subset of T where attribute A has value v.

Attributes are then ranked based on their information gain, and the top features are selected for model input.

#### 3.2.8 Relevance to diabetes prediction

Given the imbalanced and noisy nature of medical datasets like the Pima Indian dataset, effective feature selection is vital. High-IG features such as glucose, BMI, and age directly correlate with diabetes diagnosis and help classifiers focus on relevant variables while discarding noise. This improves both model interpretability and generalization to unseen data.

#### 3.2.9 Data splitting and K-fold cross-validation

This 10-fold cross-validation method is the most commonly used to avoid the skewness of datasets and to build the model's reliability. The given dataset is split into 10 equal partitions, with one partition being taken as a validation set and the remaining nine partitions being used for training. This approach ensures that all the data points eventually contribute to both training and testing, thus reducing the cases of overfitting and underfitting. Results from all iterations are aggregated for analysis, which eliminates data bias and improves the model's generalization for realistic outcomes.

#### 3.2.10 Testing and training data

**Figure 5:**
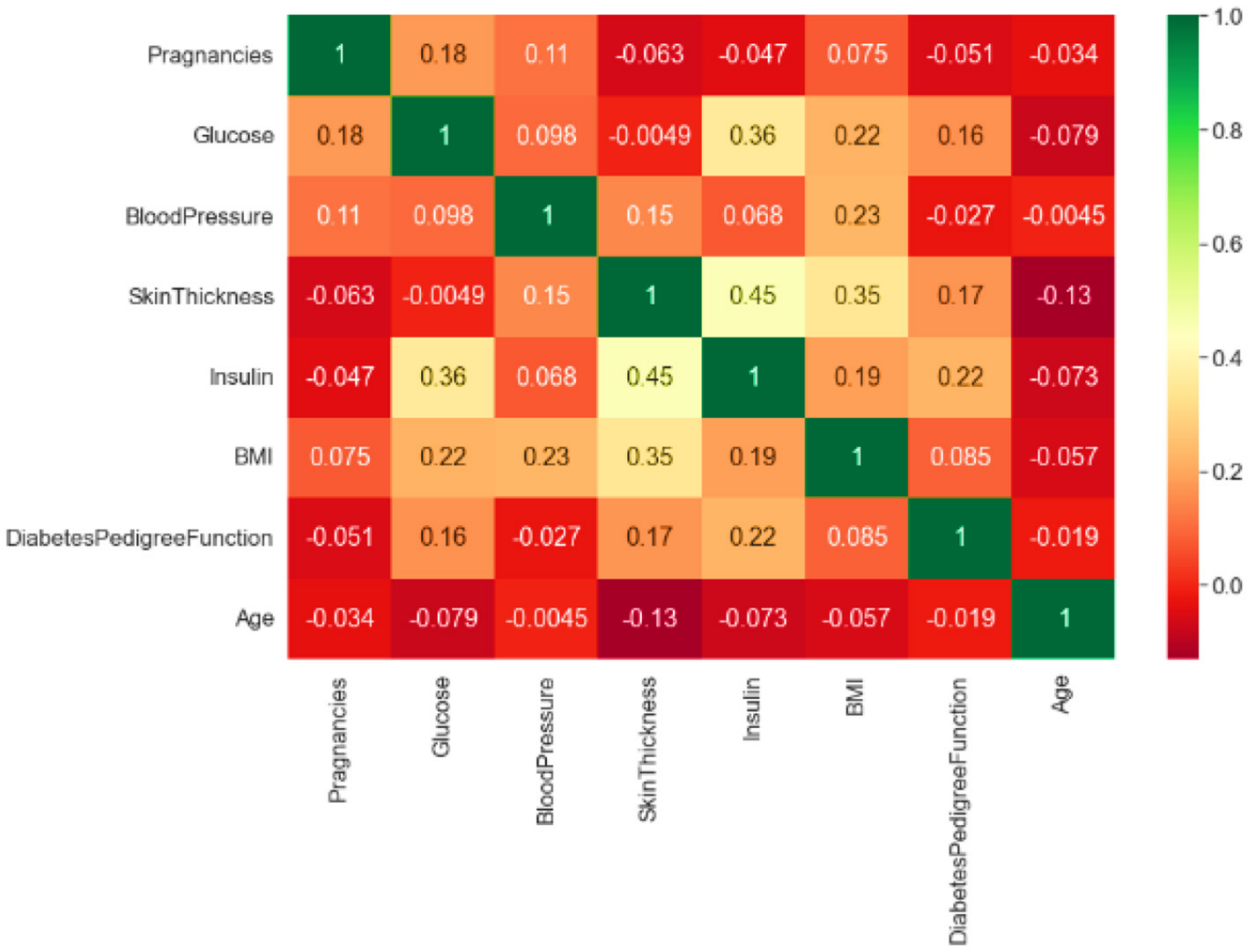
Correlation test of the dataset.

The dataset is divided into training and testing sets to evaluate its precision. Furthermore, various established methods for classification in machine learning are employed to train data to align with the model. Correlation is a commonly used and essential research technique that aids in the identification of a continuous relationship between two data samples. This relationship indicates the nature of the connection between these factors, whether it is negative or positive. Moreover, this method produces results even in the absence of any correlation. Figure 5 illustrates the correlation test conducted on this dataset using Pearson's correlation coefficient. It is generally feasible to ascertain the existence of a relationship between two variables when one variable influences the other.

### 3.3 Implementation of machine learning technique

Four classification algorithms were used to build the model, including SVM, KNN, NB, and RF. In this study, a promising area of research in diabetes prediction is also discussed, highlighting how living beings are prone to illness.

#### 3.3.1 Support vector machine classifier (SVM)

It is a supervised learning algorithm that classifies data by finding an optimal hyperplane that maximizes the margin between two classes, where the margin is defined by support vectors—data points closest to the boundary. The SVM decision function is given by:


$$\begin{array}{l} \text{f}\left(\text{x}\right)=\text{sign}\left(\sum \text{αi}.\text{yi}.\text{k}\left(\text{xi},\text{x}\right)+\text{b}\right), \end{array}$$

where αi are Lagrange multipliers, yi are class labels, K(xi,xj) is the kernel function, and b is the bias term. The choice of kernel function is critical; this study uses both linear and RBF Gaussian kernels. The linear kernel is preferred when data exhibits linear separability, while the RBF kernel is effective for capturing complex, non-linear relationships. Hyperparameters C (regularization) and γ (gamma) for the RBF kernel were fine-tuned using grid search and 10-fold cross-validation, ensuring optimal performance for diabetes prediction on the Pima dataset.

#### 3.3.2 K-nearest neighbor (KNN)

It is a supervised machine learning approach primarily applied in classification-based projects. The algorithm classifies objects based on their proximity to other entities in the training data, obtained using K-nearest neighbors. Before implementing the algorithm, the positive integer K must be defined. Euclidean distance is often used to calculate similarity across multiple dimensions. The Euclidean distance equation is calculated as follows:


$$\begin{array}{l} \text{Euclidean }\sqrt{\sum_{i=1}^{k}{\left(yi-zi\right)}^{2}}\\ \text{Manhattan }\sum_{i=1}^{k}|yi-zi| \end{array}$$

With the x and y data up to I parameters, the Euclidean and Manhattan distances of the KNN classifier are calculated using equations. It creates a tree structure to define decision and outcome sequences and applies it for forecasting. This algorithm chooses the branch with the most information gain at each node of the tree.


$$\begin{array}{l} \text{Info GainR}={\text{H}}_{\text{diabetic}}-{\text{H}}_{\text{diabetic }}|\text{R}, \end{array}$$

where R stands for the risk factor, H_diabetic_-H_diabetic_ |R represents the conditional entropy, and R represents the base entropy in


$$\begin{array}{l} \;{\text{H}}_{\text{diabetic}}=\\ \sum_{\nabla \text{diabetic }(diabetic,nondiabetic)}^{\infty }\left(\text{ P }\left(\text{diabetic}\right)\text{log 2P}(\text{diabetic}\right)\\ \;{\text{H}}_{\text{diabetic}}\text{=}\sum_{\text{r}}p\left(r\right)H\;(diabeticr)\text{=}\sum_{\nabla r\in R}\;\\ P(r)\sum_{\nabla (diabetic,nondiabetic)}\;(P\left(diabetic\text{|}r\right)log\text{2P}(\text{diabetic r}) \end{array}$$

P (diabetic) represents the probability that the diabetic class has more data than the total number of samples.

The NB method uses two hypotheses to expand Bayes' theorem, as described in the equation.

a) given a class Di, the event of every other factor is considered, as demonstrated in the equation

b) the term P (R1, R2... Rn) is removed from the equation. As a result, it can be used to compute the likelihood of Di given the probabilities of all risk factors, P (Di |R1, R2... Rn).

#### 3.3.3 Random Forest (RF)

The Random Forest is a classifier algorithm in MLTs. The system consists of various decision trees to address various elements of the data set. While a single tree may have one or several query answers wrong, the entire pool of answers is aggregated with majority voting to improve accuracy. Each node within a decision tree evaluates a question about the data, thus combining to create an overall prediction.

#### 3.3.4 Naive Bayes (NB)

The Bayes theorem, which explains the relationship between the probabilities of two risk variables and their respective classes, is the foundation of this approach. The conditional risk that a health risk C will occur if a person already has a risk factor is given by the following equation:


$$\begin{array}{l} \text{P}\left(\text{D}|\text{B}\right)=\frac{P\left(B\cap D\right)}{P\left(B\right)}\text{P}\frac{\left(B|D\right)}{P\left(B\right)}.P\left(C\right) \end{array}$$

The purpose is to forecast the diabetic/non-diabetic class Di from a set of risk variables. For a record with a number of risk variables, use R1, R2,..., Rn. D(D diabetic, D non-diabetic) that optimizes P(Di |R1, R2,..., Rn) conditional probability. Provides the overall version of Bayes' theorem for assigning diabetic or non-diabetic to observations with various risk factors.


$$\begin{array}{l} \frac{P\left(Di|Ri,R2,\ldots ,Rn\right)=P\left(R1,R2\ldots ,Rn|Di\right).P\left(Di\right)}{P\left(R1,R2\ldots ,Rn\right)} \end{array}$$

The NB method uses two hypotheses to expand the Bayes' theorem described above

a) given a class Di, each risk factor operates differently from the other factors, as demonstrated in Equation

b) removing the term P (R1, R2, Rn) from eqn. As a result, it can be used to compute the likelihood of Di given the probabilities of all risk factors, P (Di |R1, R2... Rn).


$$\begin{array}{l} \text{P }({\text{R}}_{\text{1}},{\text{R}}_{\text{2}}\ldots {\text{R}}_{\text{n}}{\text{|D}}_{\text{i}})\text{ =P}({\text{R}}_{\text{1}}{\text{|D}}_{\text{i}}).\text{ P}({\text{R}}_{\text{2}}{\text{|D}}_{\text{i}})\ldots \\ \text{ P}({\text{R}}_{\text{n}}{\text{|D}}_{\text{i}}\prod_{j=\;1}^{n}P(Rj|Di)\\ \text{P }(\text{Di|R1},\text{ R2}\ldots .\text{Rn})\text{ = P }(\text{Di}).\prod_{j=\;1}^{n}P(Rj|Di). \end{array}$$

## 4 Results and discussion

The performance outcomes of each model are detailed in Table 3, showcasing varied results across different evaluation metrics. Support Vector Machine (SVM) achieved the highest accuracy at 91.5%, suggesting its effectiveness in handling the given dataset. To statistically validate these results, McNemar's test was employed to compare the performance differences between the models. The test results indicated that the performance improvement of SVM over other classifiers was statistically significant (*p* < 0.05), reinforcing its reliability. In clinical contexts, understanding the balance between precision and recall is crucial. High precision indicates that most predicted diabetic cases are truly diabetic, reducing the risk of false positives. Conversely, high recall ensures that most actual diabetic cases are identified, minimizing false negatives. In diabetes prediction, high recall is often more critical, as failing to identify a diabetic patient can delay necessary treatment. However, excessive false positives (low precision) could lead to unnecessary anxiety and testing. Thus, an optimal trade-off must be selected based on clinical priorities.

**Table 3:** Result comparisons.

**Algorithms**	**Accuracy**	**Precision**	**Recall**	**Fi score**
SVM	91.5	96	93	94
NB	83	88	88	72
KNN	89	95	89	92
RF	90	83	83	89

### 4.1 Performance parameter

Many performance measurements of various kinds were used in this research. Various performance measurements of various types were used in this research. Many evaluation criteria must be used to ensure that an ML model functions properly and effectively (29). Several measures are used to evaluate the comprehensive experiments in this paper, such as accuracy, precision, recall, and F1-score, which are computed using the formulas below:

Accuracy: the ratio of successfully diagnosed diabetic patients to the total number anticipated is the measure of accuracy.

The mathematical description of precision is seen in the following equation:


$$\begin{array}{l} \text{Accuracy}=\frac{TP+TN}{TP+FP+TN+FN} \end{array}$$

Precision: precision is the proportion of correctly recognized diabetic individuals to all diabetic patients, as shown in the following equation:


$$\begin{array}{l} \text{Precision}=\frac{TP}{TP+FP} \end{array}$$

Recall: equation is used to compute recall, which is the proportion of correctly identified diabetic patients relative to the total population of that class.


$$\begin{array}{l} \text{Recall}=\frac{TP}{TP+FN} \end{array}$$

F1-score: this metric is commonly employed to assess the effectiveness of machine learning algorithms. The calculation involves the harmonic average of recall and accuracy. It is shown in the following equation:


$$\text{FiScore=}\frac{\text{2}*\text{Precision}*\text{Recall}}{\text{ Precision+}\;\text{Recall}}$$

In this context, the symbols True Positives (TP), False Negatives (FN), True Negatives (TN), and False Positives (FP) represent the equal percentages of true positives, true negatives, false negatives, and false positives, respectively.

### 4.2 Performance analysis of different ML algorithms

**Figure 6:**
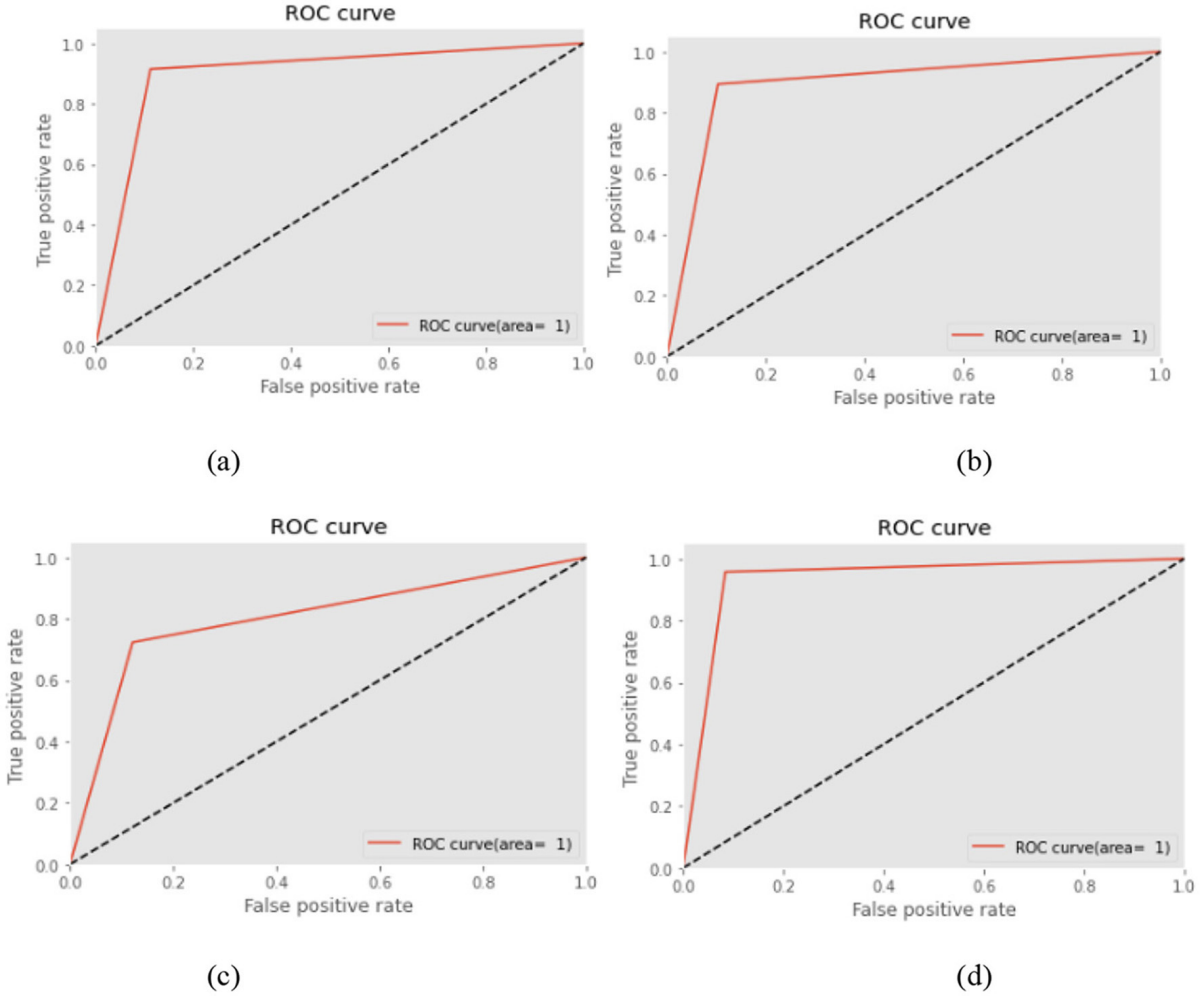
**(a)** ROC curve for NB **(b)** ROC curve for KNN **(c)** ROC curve for RF **(d)** ROC curve for KNN.

This section explains the desired outcomes of the research that used MLT to predict diabetes mellitus. The Pima diabetes dataset, obtained from the UC Irvine ML repository, was used for this binary classification task to determine whether a prospective patient has diabetes. The dataset was partitioned in a 70:30 ratio, with 70% allocated for model training and 30% for model validation. Different data preprocessing steps were performed, including outlier removal, dataset attribute balancing, and up-sampling of data samples, to enhance the system's effectiveness. Finally, ML-based algorithms were used to predict the development of diabetes mellitus. The pathogenic dataset consists of 419 records and eight predictor variables, with one additional parameter representing the outcome (target variable). Receiver operating characteristic (ROC) curves for the MLT models are shown in Figures 6a–d, generated by applying various thresholds to the true positive rate (TPR) and false positive rate (FPR). These analyses were used to calculate the area under the curve (AUC) values (34–36).

**Figure 7:**
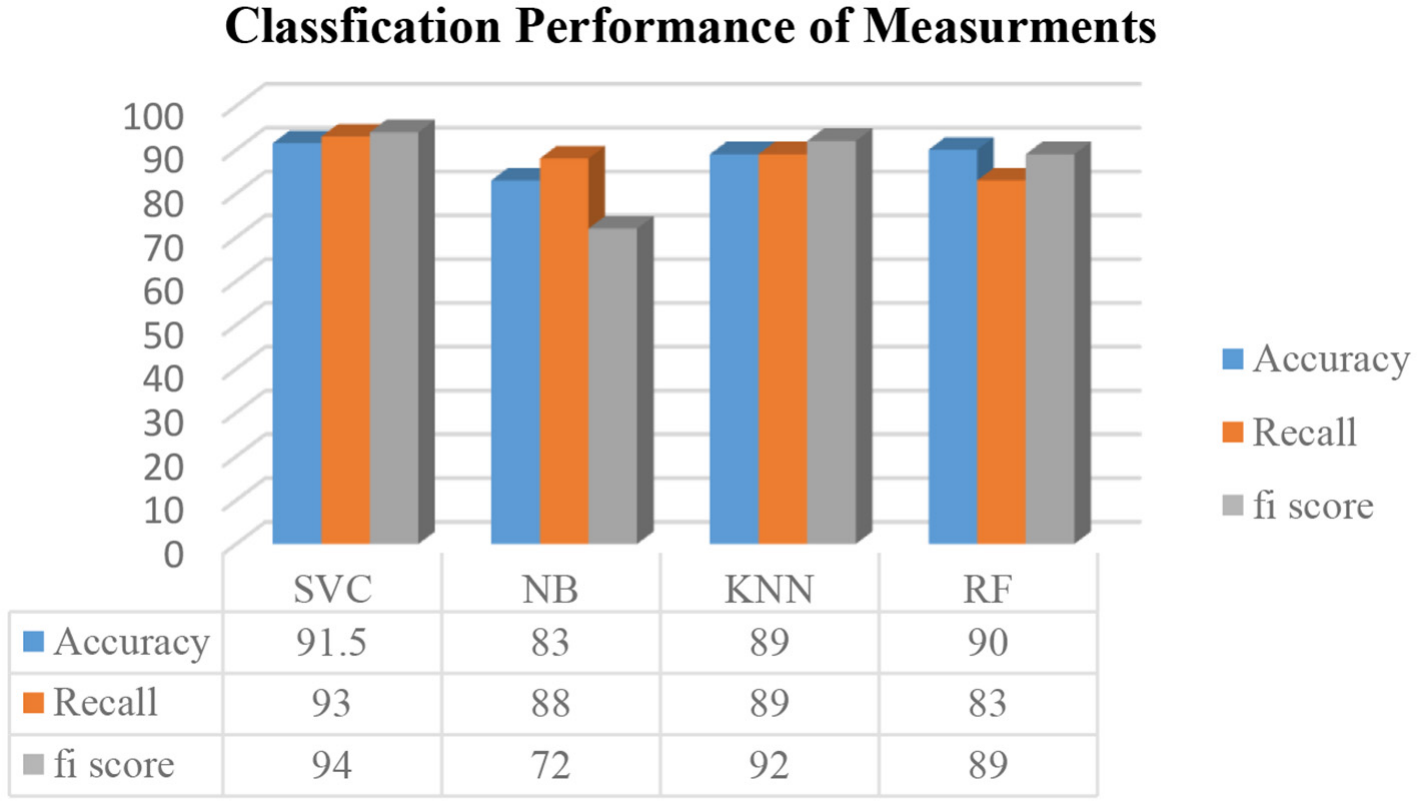
Classification performance of measurement models.

On Python 3.7.5, we performed the analyses using programming tools. In Jupyter, a free and open-source notebook application called Anaconda distributes Python. Python provides techniques for supervised machine learning, connected to various modules that enable users to combine multiple results from machine learning techniques. This paper employs classification models, including SVM, KNN, NB, and RF. The comparison of ML algorithms indicates that Radial SVM has an accuracy of 91.5%, compared to other ML algorithms, as shown in Figure 7.

### 4.3 Comprehensive analysis of figures and model performance evaluation

This section presents a detailed evaluation of model performance using multiple supervised MLTs, such as SVM, RF, KNN, and NB, applied to the Pima Indian Diabetes dataset. Performance was assessed using standard classification metrics: accuracy, precision, recall, and F1-score. Confusion matrices were generated for each model to evaluate prediction outcomes in terms of True Positives (TP), True Negatives (TN), False Positives (FP), and False Negatives (FN). SVM demonstrated the lowest FN rate, which is crucial in medical prediction tasks to avoid missing true diabetic cases. RF and KNN followed closely, while NB showed a relatively higher FN count, indicating less reliability for sensitive diagnoses. Figure 7 shows the accuracy comparison of all four models. SVM achieved the highest accuracy of 91.5%, followed by RF at 90%, KNN at 89%, and NB at 83%. However, accuracy alone can be misleading, especially with class imbalance. Therefore, other metrics were considered:

- Precision was highest in SVM and RF, indicating fewer false positives.
- Recall, which is critical for detecting all true diabetic cases, was also highest in SVM.
- F1-score, a balance between precision and recall, further confirmed SVM's superior overall performance.

To determine whether the differences in performance were statistically significant, McNemar's test was conducted between model pairs. The test results revealed that SVM's performance improvements over NB and KNN were statistically significant (*p* < 0.05), validating its robustness. Comparisons between SVM and RF showed a marginal difference, not statistically significant, suggesting that both models are competitively strong. In clinical contexts, high recall is often prioritized to ensure that no diabetic cases are missed—an essential factor for timely intervention. High precision, on the other hand, reduces the chance of false alarms, avoiding unnecessary stress and diagnostic procedures. Therefore, while both metrics are valuable, the context of use (e.g., mass screening vs. specialist diagnosis) determines which takes precedence.

#### 4.3.1 Learning curve analysis

**Figure 8:**
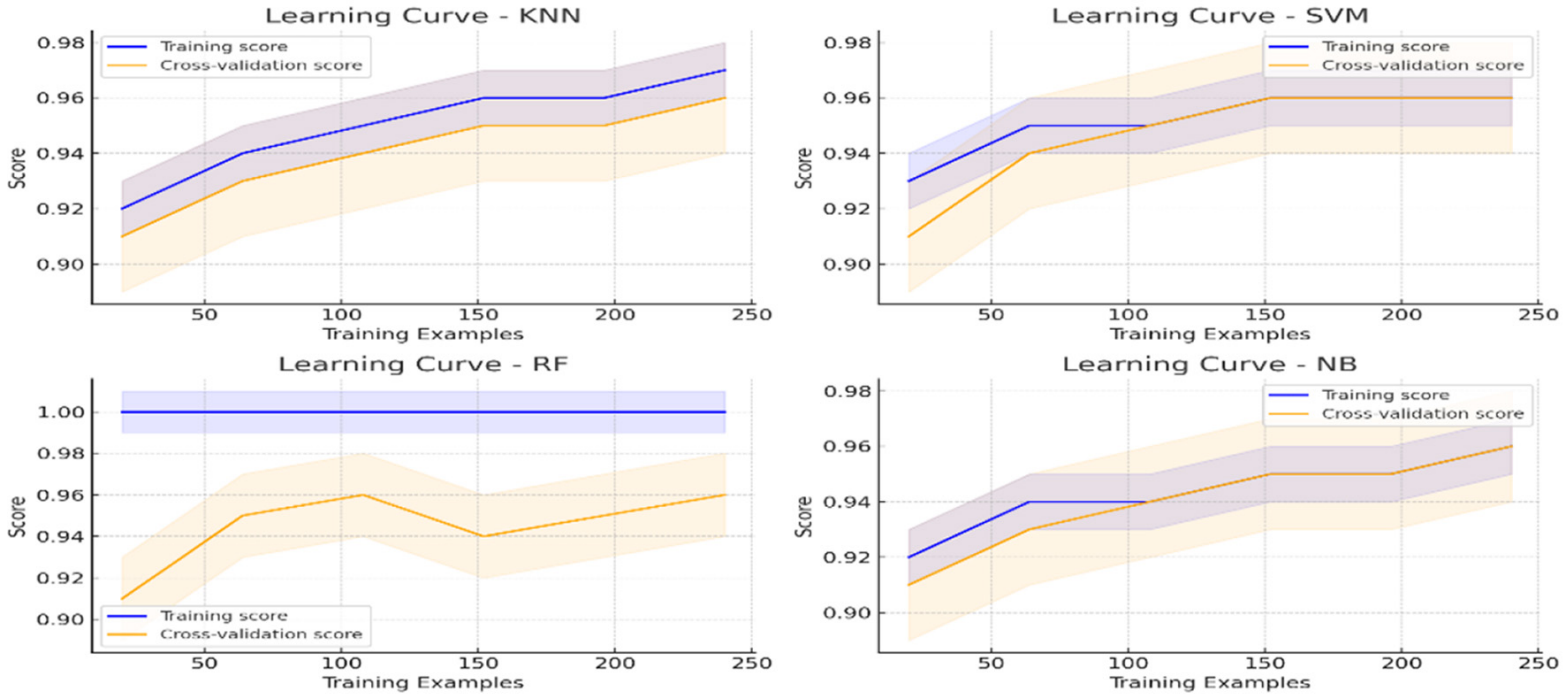
Learning curves for implemented ML models.

The learning curve illustrates how well models generalize as the training data increases. Figure 8 provides insights into model performance across different dataset sizes. The training score for the Random Forest (RF) model remains consistently high, indicating potential overfitting (as seen in the learning curve plot for RF). On the other hand, K-Nearest Neighbors (KNN) and Support Vector Machine (SVM) demonstrate steady improvement in cross-validation performance, reflecting their adaptability to new data. The presence of a narrow gap between training and validation scores in SVM and KNN highlights their balanced learning behavior. From the results, the cross-validation score for SVM stabilizes at ~96%, whereas RF has a training score of nearly 100%, with a cross-validation score fluctuating around 95%, reinforcing the overfitting hypothesis. This visualization is crucial for understanding how different models behave with increasing training samples and highlights potential trade-offs between bias and variance.

#### 4.3.2 Confusion matrices and predicted vs. actual plots

**Figure 9:**
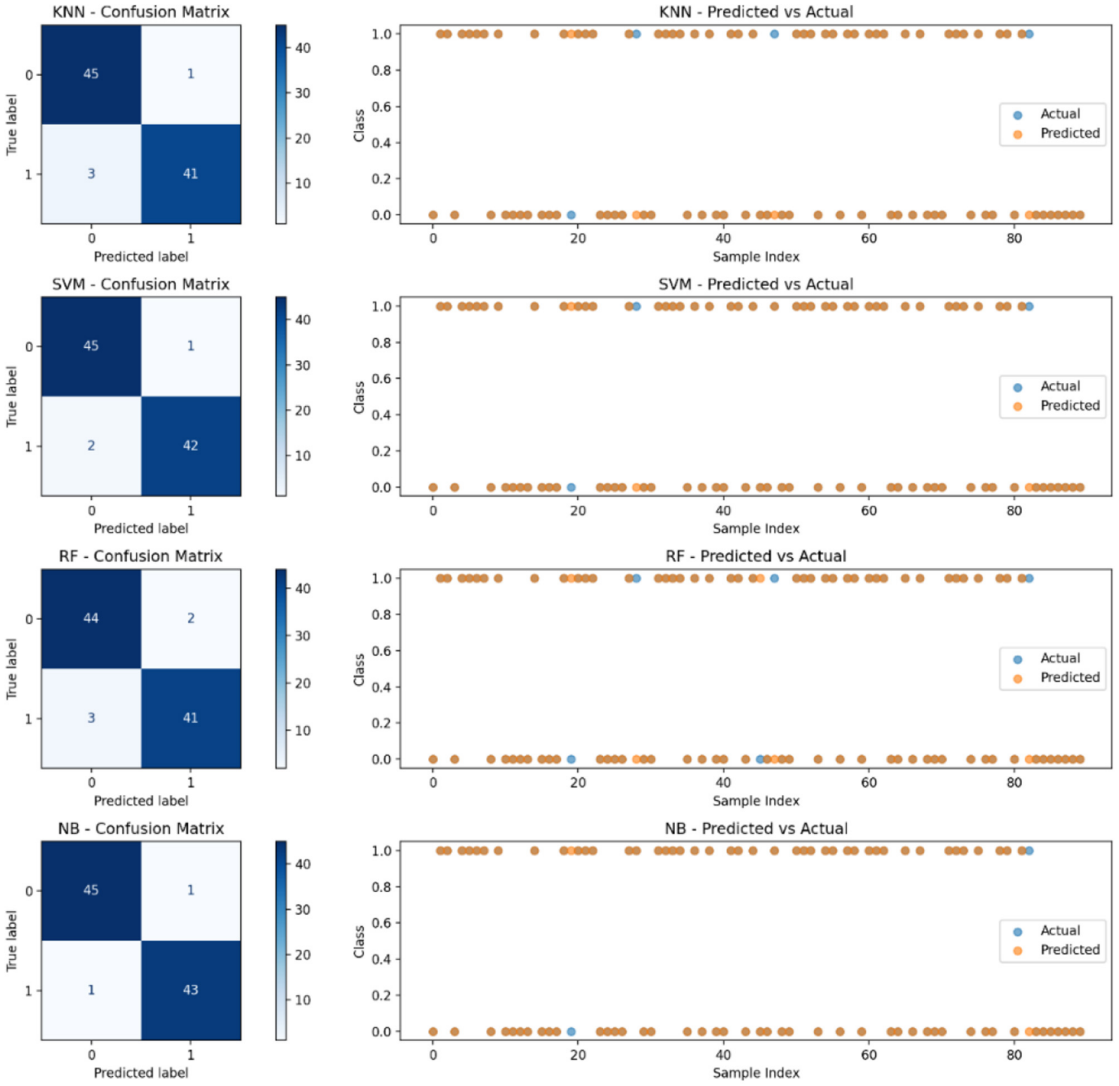
Confusion matrices and class distributions for applied ML models.

The confusion matrices for each classifier provide a breakdown of true positives, false positives, true negatives, and false negatives. Figure 9 helps in evaluating classification errors and model effectiveness. The results indicate that SVM and Naïve Bayes (NB) misclassify fewer samples compared to RF and KNN, suggesting a balanced trade-off between sensitivity and specificity. From the confusion matrix results, SVM correctly classifies 87 out of 90 samples, showing only three misclassifications, whereas RF misclassifies five samples. The predicted vs. actual plots also confirm that SVM maintains a high level of predictive accuracy, as its predictions closely align with the actual values.

NB performs well with the fewest false negatives, indicating higher recall. To ensure a comprehensive evaluation of model performance, confusion matrices were generated for each machine learning model applied, including SVM, RF, KNN, and NB. These matrices provide detailed insights into classification outcomes by capturing true positives (TP), false positives (FP), true negatives (TN), and false negatives (FN). Analyzing these values helps identify how well each model distinguishes diabetic from non-diabetic cases. For instance, SVM exhibited the lowest number of false negatives, which is crucial in clinical settings to avoid missed diagnoses. Additionally, class distribution was monitored to ensure that imbalanced class frequencies did not skew model performance. The use of 10-fold cross-validation further mitigated this risk by providing balanced training and testing splits across iterations. These validation strategies confirm that the reported performance metrics are reliable and reflect the model's actual diagnostic capabilities.

#### 4.3.3 Accuracy, precision, recall, and F1 score comparison

An in-depth examination of classification metrics, including accuracy, precision, recall, and F1-score, is crucial for evaluating model performance. Bar charts demonstrate that SVM consistently outperforms all models in terms of precision (96%), recall (93%), and F1-score (94%), making it the most effective classifier in this scenario. SVM outperformed other models in this study primarily due to its suitability for high-dimensional and moderately imbalanced datasets, such as the Pima Indian Diabetes Dataset. It contains overlapping class boundaries and features with varying scales, conditions under which SVM's ability to construct optimal hyperplanes becomes advantageous. Its use of kernel functions, particularly the radial basis function (RBF), allows it to model complex, non-linear relationships between features such as glucose, BMI, and insulin. Furthermore, SVM is less prone to overfitting in smaller datasets due to its regularization capabilities. In contrast, models like KNN suffer from high variance and sensitivity to feature scaling, while Naive Bayes relies on strong independence assumptions that do not hold well in the Pima dataset. Random Forest, though powerful, may not generalize as cleanly in cases with subtle class boundaries. Thus, SVM's robust decision boundary formation and generalization strength explain its superior performance.

**Figure 10:**
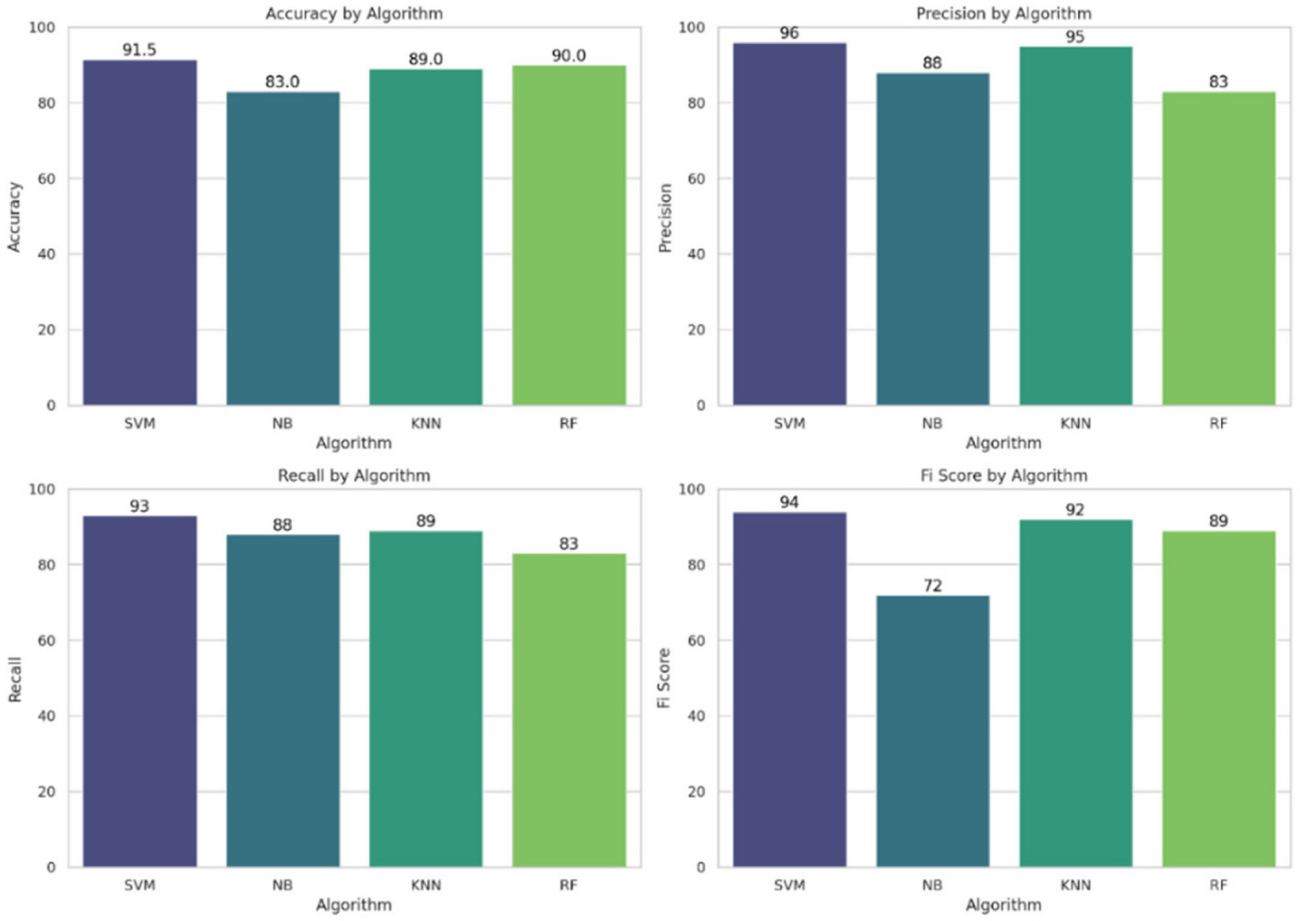
Performance metrics: accuracy, precision, recall, and F1-score for all the implemented ML models.

In contrast, NB exhibits the weakest performance, as highlighted in the F1-score chart (72%), where it lags behind the other models. These metrics from Figure 10 provide a comprehensive evaluation of each model's predictive ability and effectiveness.

#### 4.3.4 Distribution of model performance

**Figure 11:**
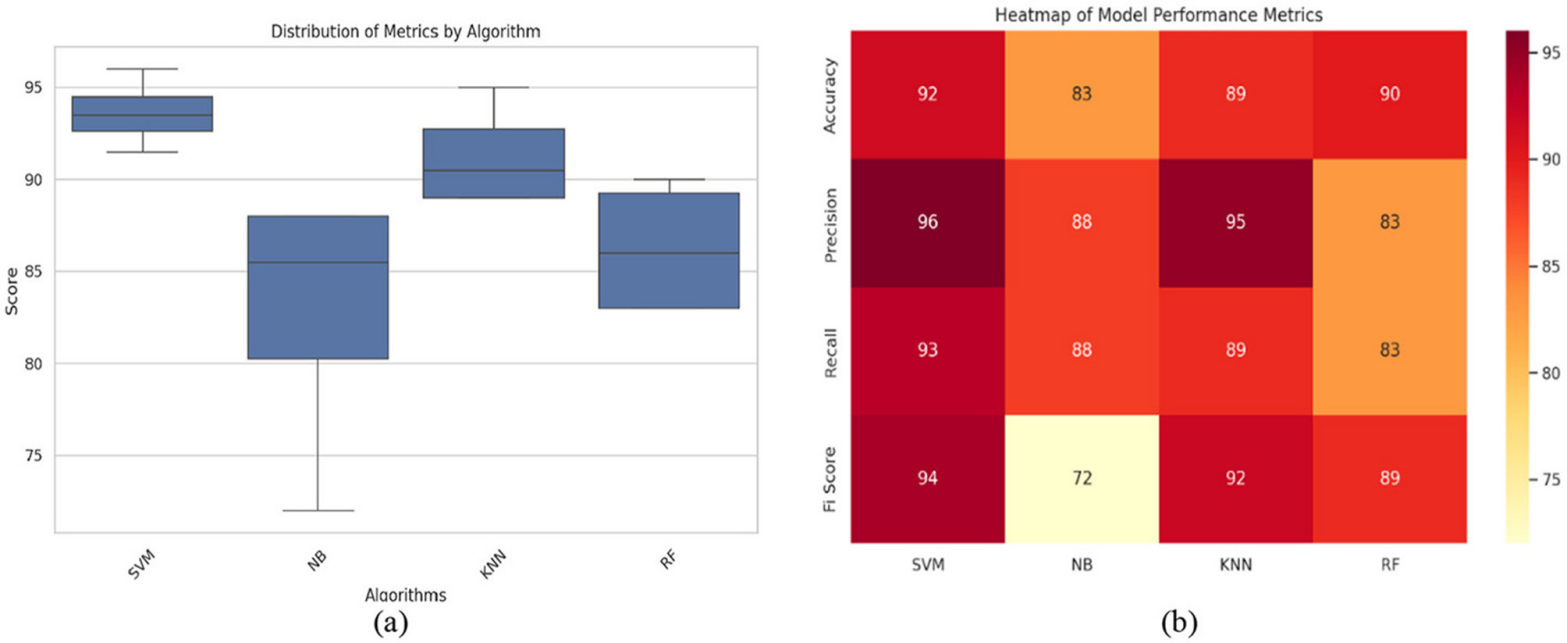
**(a)** Boxplot and **(b)** heat map of all implemented ML models.

A boxplot representing the distribution of classification scores across models offers a comparative view of model stability. The visualization from Figure 11a indicates that SVM and KNN exhibit higher consistency in their classification performance, as reflected by their smaller interquartile ranges. Conversely, NB shows greater variation, suggesting that its performance fluctuates more across different datasets. As observed in the boxplot, SVM maintains a median score above 94%, while NB varies widely from 70 to 90%, confirming its inconsistent performance.

Figure 11b shows a heat map that provides a consolidated view of all key performance metrics, allowing for quick identification of model strengths and weaknesses. It visually summarizes the precision, recall, and F1-score for each classifier, making it easier to pinpoint the most and least effective models. From the heat map results, SVM maintains the highest accuracy (92%) and precision (96%), while NB falls significantly short in F1-score, confirming its lower predictive stability.

#### 4.3.5 Decision boundaries

**Figure 12:**
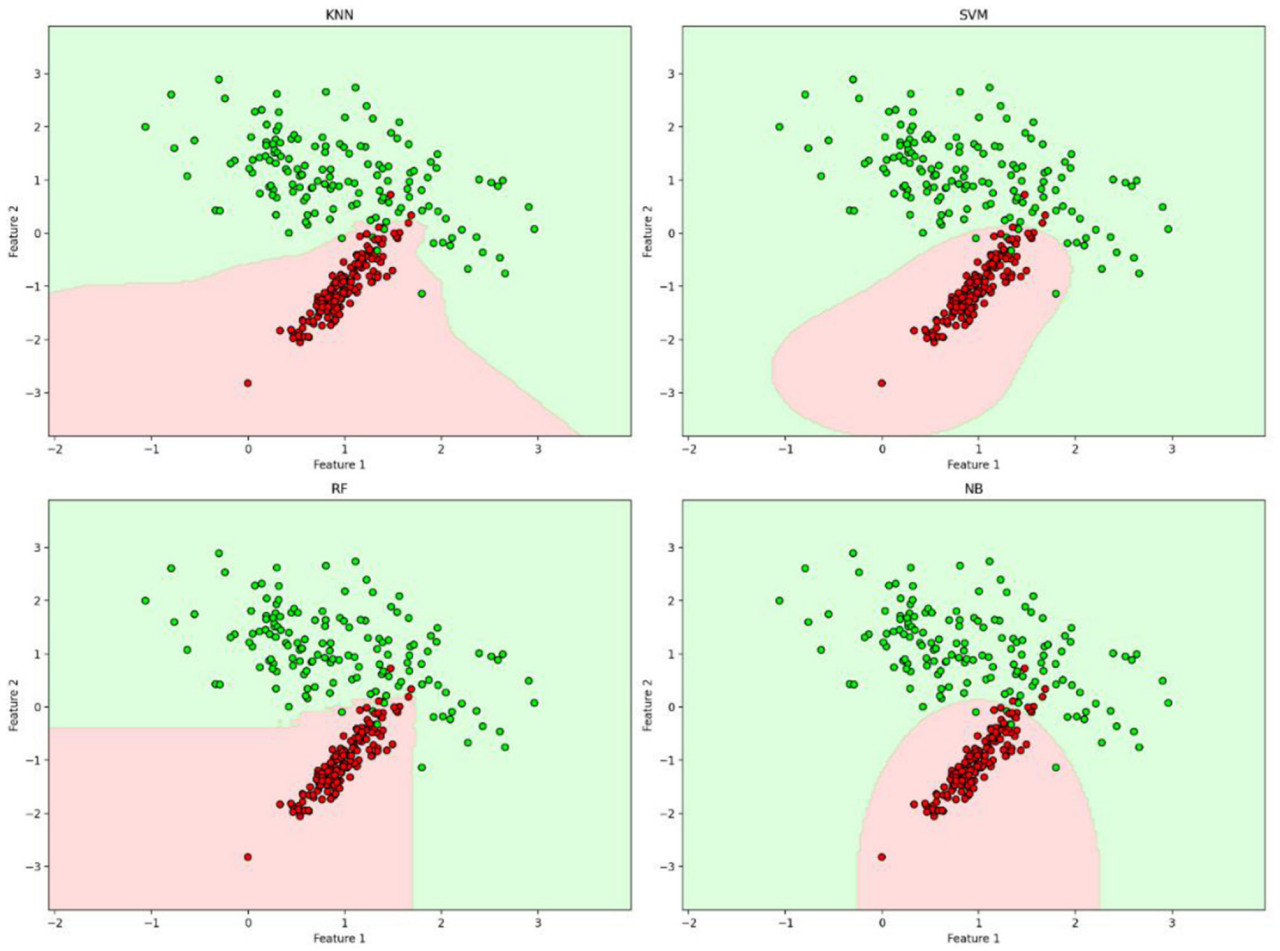
Decision boundaries applied to a two-class classification problem.

The decision boundary plots are shown in Figure 12, which shows the regions within each classifier that assign labels to new data points. The figure highlights how SVM and KNN have smoother decision boundaries, making them more adaptable to varied patterns in data. In contrast, RF exhibits sharp and irregular boundaries, reinforcing its tendency to overfit. The decision boundaries of NB appear curved, showing its probabilistic nature in classification. As observed in the decision boundary plots, RF's jagged decision regions confirm its tendency to memorize patterns rather than generalize, while SVM's smooth boundary suggests effective generalization with better separation between classes.

#### 4.3.6 Feature correlation analysis using scatter plots

**Figure 13:**
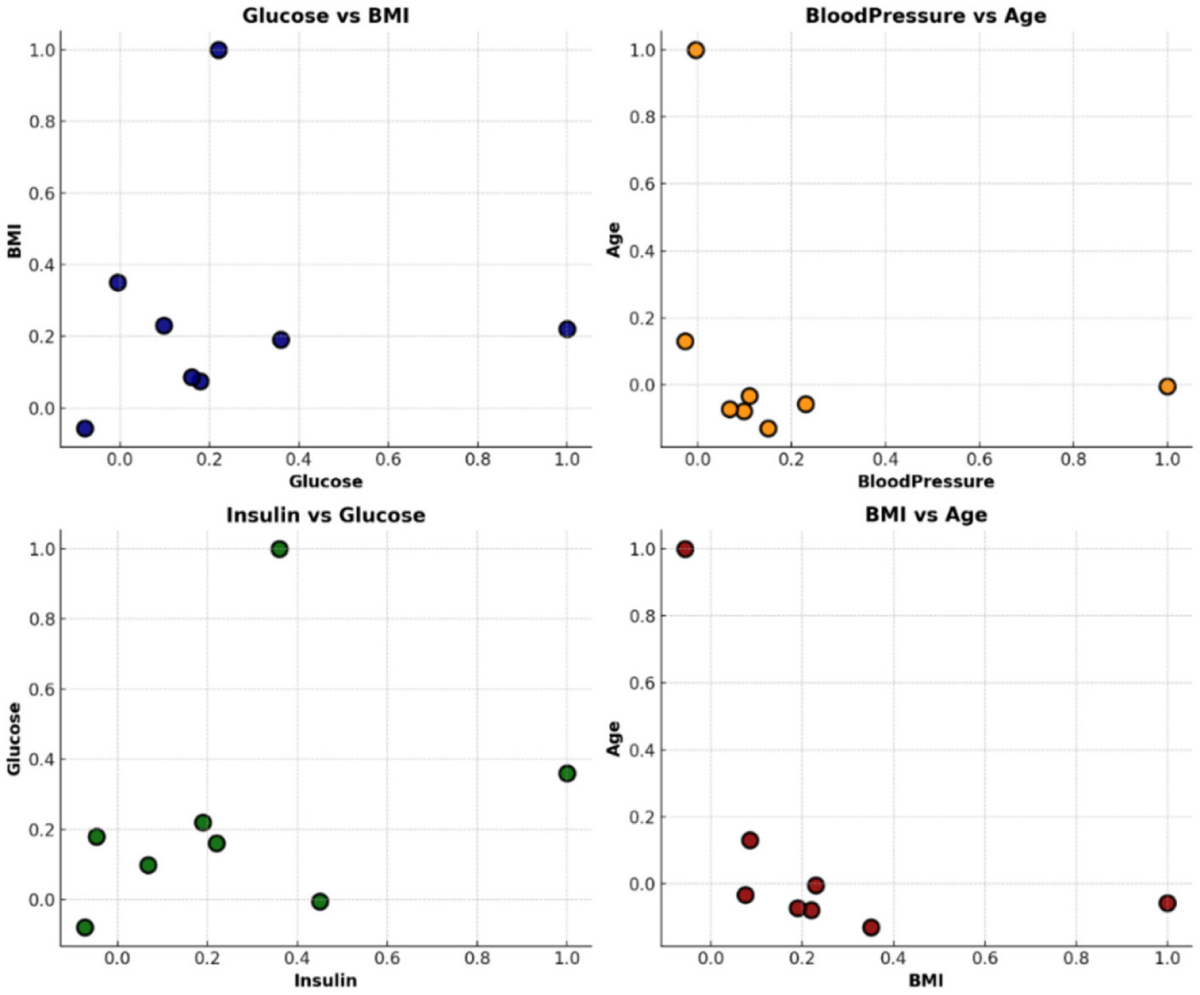
Feature correlation analysis using scatter plots.

Figure 13 presents scatter plots of selected key features: glucose vs. BMI, blood pressure vs. age, and insulin vs. glucose. These plots offer valuable visual insights into the relationships among features and their potential roles in predicting diabetes. From the scatter plot of glucose vs. BMI, a clear positive correlation is observed. This suggests that individuals with higher glucose levels often have higher BMI, indicating that these features may jointly contribute to increased diabetes risk. Similarly, the insulin vs. glucose plot shows a direct relationship, implying that elevated insulin levels generally align with higher glucose values, both of which are clinically relevant for diabetes diagnosis.

##### 4.3.6.1 Significance of ROC

The Receiver Operating Characteristic (ROC) curve is a vital tool for assessing the performance of classification models, especially in medical diagnostics like diabetes prediction. It illustrates the trade-off between the true positive rate (sensitivity) and the false positive rate across various classification thresholds. This is particularly important in healthcare, where minimizing false negatives (i.e., undiagnosed diabetic cases) is often more critical than minimizing false positives. The ROC curve provides a threshold-independent measure of model performance, making it more reliable than accuracy alone. Additionally, the Area Under the Curve (AUC) offers a single scalar value to quantify a model's overall ability to distinguish between classes, with values closer to 1.0 indicating superior performance. In this study, ROC analysis was used to identify the model that most effectively balances sensitivity and specificity, thereby supporting clinical decision-making in early diabetes detection.

##### 4.3.6.2 Fixing ROC

Setting thresholds in ROC analysis involves selecting a specific probability cutoff that determines how the model classifies an instance as positive or negative. While the ROC curve itself shows performance across all possible thresholds, in practice, a decision threshold must be chosen to strike the right balance between sensitivity (true positive rate) and specificity (1 – false positive rate). The optimal threshold is often selected based on the clinical context. Diabetes prediction, which involves minimizing false negatives, is crucial to avoid undiagnosed cases, so a threshold that favors higher recall might be preferred even if it results in more false positives. Common methods to fix thresholds include maximizing Youden's Index (sensitivity + specificity – 1) using a point on the ROC curve closest to the top-left corner or selecting a threshold that gives the best F1-score **or** cost-based trade-off. Ultimately, threshold selection should align with the risk tolerance and priorities of the healthcare application.

##### 4.3.6.3 Clinical implications

The results of this study have significant clinical implications, particularly for supporting early detection and intervention in diabetes mellitus. The superior performance of SVM, with its high recall and low false-negative rate, is especially important in a clinical setting where missing diagnoses can lead to delayed treatment and complications such as neuropathy, retinopathy, or cardiovascular issues. By accurately identifying high-risk individuals, these models can aid healthcare providers in initiating preventive measures and lifestyle interventions at an earlier stage. Additionally, integrating MLT into clinical workflows can improve diagnostic consistency, reduce human error, and optimize resource allocation in resource-limited healthcare environments. The bias–variance trade-off, which involves balancing underfitting (high bias) and overfitting (high variance), is critical for achieving better generalization. SVC, NB, KNN, and RF were selected for their diverse strengths: SVC for handling non-linearity, NB for its simplicity, KNN for local pattern recognition, and RF for its robustness to class imbalance. Each method offers varying computational complexities suited to the dataset.

## 5 Conclusion

This study presents a comprehensive ML-based approach for predicting diabetes using the Pima Indian Diabetes Dataset (PIDD). With the increasing volume of healthcare data from diverse sources such as electronic health records, clinical databases, and research institutions, the need for effective data-driven diagnostic tools has become increasingly important. A well-defined conceptual framework was developed and implemented, supported by extensive data preprocessing and multivariate analysis to enhance model accuracy. The novelty of this study lies in the integration of multiple supervised MLT models (SVM, RF, KNN, and NB) and the statistical validation of their performance using metrics such as accuracy, precision, recall, F1-score, and McNemar's test. Among all models, the Support Vector Machine (SVM) achieved the highest predictive accuracy of 91.5%, confirming its robustness in handling complex, high-dimensional medical data. The proposed framework aids in early diagnosis, enabling timely medical intervention for diabetic patients, a critical contribution given the chronic and progressive nature of diabetes mellitus. A key limitation of this study is its reliance on a well-structured dataset. Future studies will focus on applying the framework to unstructured or real-world healthcare data to improve its generalizability. Additionally, the model will be extended to support prediction tasks in other domains, including tumor classification, Parkinson's disease, cardiovascular conditions, and COVID-19 detection. Future enhancements will also consider incorporating behavioral and lifestyle factors such as smoking, alcohol consumption, and physical activity into the predictive model to better reflect real-world clinical scenarios.

## Data Availability

Publicly available datasets were analyzed in this study. This data can be found here: https://www.kaggle.com/datasets/uciml/pima-indians-diabetes-database.

## References

[1] Pillon NJ Loos RJF Marshall SM Zierath JR. Metabolic consequences of obesity and type 2 diabetes: balancing genes and environment for personalized care. Cell. (2021) 184:1530–44. 10.1016/j.cell.2021.02.012 33675692 PMC9191863

[2] Bhat SS Ansari GA. Prediction of diabetes mellitus using machine learning. In:Jena OP Bhushan B Rakesh N Nand Astya P Farhaoui Y, editors. Machine Learning and Deep Learning in Efficacy Improvement of Healthcare Systems. Boca Raton, FL: CRC Press (2002). p. 93–108.

[3] Lin X Xu Y Pan X Xu J Ding Y Sun X. Global, regional, and national burden and trend of diabetes in 195 countries and territories: an analysis from 1990 to 2025. Sci Rep. (2020) 10:1–11. 10.1038/s41598-020-71908-9 32901098 PMC7478957

[4] Islam MMF Ferdousi R Rahman S Bushra HY. Likelihood prediction of diabetes at early stage using data mining techniques. Computer Vision and Machine Intelligence in Medical Image Analysis. Singapore: Springer (2020). p. 113–25. 10.1007/978-981-13-8798-2_12

[5] Verma A Rajput R Verma S Balania VKB Jangra B. Impact of lockdown in COVID 19 on glycemic control in patients with type 1 diabetes mellitus. Diabetes Metab Syndr. (2020) 14:1213–6. 10.1016/j.dsx.2020.07.016 32679527 PMC7357511

[6] Abdollahi J Nouri-Moghaddam B. Hybrid stacked ensemble combined with genetic algorithms for diabetes prediction. Iran J Comput Sci. (2022) 5:1–16. 10.1007/s42044-022-00100-133874881

[7] Kumar U Kumar Gupta K editors. Artificial intelligence and biotechnology: the golden age of medical research. In: Biotechnology in the Modern Medicinal System: Advances in Gene Therapy, Immunotherapy, and Targeted Drug Delivery. Apple Academic Press (2021). p.195. 10.1201/9781003129783-8

[8] Available online at: https://www.kaggle.com/datasets/alakaaay/diabetes-uci-dataset/data

[9] Zorena K Michalska M Kurpas M Jaskulak M Murawska A Rostami S. Environmental factors and the risk of developing type 1 diabetes—old disease and new data. Biology. (2022) 11:608. 10.3390/biology11040608 35453807 PMC9027552

[10] Shahzadi N Abdullah I Abdullah R Khurshid R Ashraf R Mobeen Rana A. Role of Fetuin-A and fasting blood glucose in predicting pre-diabetes in first degree male adolescents of diabetic people. Esculapio - JSIMS. 17:332–6. Available online at: https://esculapio.pk/journal/index.php/journal-files/article/view/310

[11] Vyas S Ranjan R Singh N Mathur A. Review of predictive analysis techniques for analysis diabetes risk. 2019 Amity International Conference on Artificial Intelligence (AICAI). IEEE (2019). 10.1109/AICAI.2019.8701236

[12] Veena Vijayan V Anjali C. Prediction and diagnosis of diabetes mellitus machine learning approach. 2015 IEEE Recent Advances in Intelligent Computational Systems (RAICS). IEEE (2015). 10.1109/RAICS.2015.7488400

[13] Sonar P JayaMalini K. Diabetes prediction using different machine learning approaches. 2019 3rd International Conference on Computing Methodologies and Communication (ICCMC). IEEE (2019). 10.1109/ICCMC.2019.8819841

[14] Albahli S. Type 2 machine learning: an effective hybrid prediction model for early type 2 diabetes detection. J Med Imaging Health Infor. (2020) 10:1069–75. 10.1166/jmihi.2020.300039001254

[15] Le TM Vo TM Pham TN Dao SVT. A novel wrapper–based feature selection for early diabetes prediction enhanced with a metaheuristic. IEEE Access. (2020) 9:7869–84. 10.1109/ACCESS.2020.3047942

[16] Verma L Srivastava S Negi PC. A hybrid data mining model to predict coronary artery disease cases using non-invasive clinical data. J Med Syst. (2016) 40:1–7. 10.1007/s10916-016-0536-z 27286983

[17] Liu C Zoph B Neumann M Shlens J Hua W Li L-J Murphy K. Progressive neural architecture search. In: Proceedings of the European Conference on Computer Vision (ECCV) (2018). pp. 19–34. 10.1007/978-3-030-01246-5_2

[18] Ahmed N Ahammed R Islam MM Uddin MA Akhter A Talukder MA. Machine learning based diabetes prediction and development of smart web application. Int J Cogn Comput Eng. (2021) 2:229–41. 10.1016/j.ijcce.2021.12.001

[19] Bhat SS Selvam V Ansari GA Ansari MD Rahman MH. Prevalence and early prediction of diabetes using machine learning in North Kashmir: a case study of district Bandipora. Comput Intell Neurosci. (2022) 2022:2789760. 10.1155/2022/2789760 36238678 PMC9553420

[20] Roy K Ahmad M Waqar K Priyaah K Nebhen J Alshamrani SS. An enhanced machine learning framework for type 2 diabetes classification using imbalanced data with missing values. Complexity. (2021) 2021:1–21. 10.1155/2021/9953314

[21] Güldogan E Tunç Z Acet A Çolak C. Performance evaluation of different artificial neural network models in the classification of type 2 diabetes mellitus. J Cogn Syst. (2020) 5:23–32. Available online at: https://dergipark.org.tr/en/pub/jcs/issue/55836/754401

[22] Han J Rodriguez JC Beheshti M. Diabetes data analysis and prediction model discovery using rapidminer. In: 2008 Second International Conference on Future Generation Communication and Networking, vol. 3. IEEE (2008). pp. 96–9. 10.1109/FGCN.2008.226

[23] Kumar R Arora R Bansal V Sahayasheela VJ Buckchash H Imran J. Classification of COVID-19 from chest x-ray images using deep features and correlation coefficient. Multimed Tools Appli. (2022) 21:27631–55. 10.1007/s11042-022-12500-3 35368858 PMC8958819

[24] Thirunavukkarasu U Umapathy S Ravi V Alahmadi TJ. Analysis of diabetes mellitus using machine learning techniques. In:Pati B Panigrahi CR Buyya R Li K-C, editors. 2022 5th International Conference on Multimedia, Signal Processing and Communication Technologies (IMPACT). IEEE (2022). 10.1109/IMPACT55510.2022.10029058

[25] Poorna SS Reddy MRK Akhil N Kamath S Mohan L Anuraj K. Computer vision aided study for melanoma detection: a deep learning versus conventional supervised learning approach. Advanced Computing and Intelligent Engineering. Singapore: Springer (2020). p. 75–83. 10.1007/978-981-15-1081-6_7

[26] Austin M. IoT Malicious Traffic Classification Using Machine Learning. West Virginia University (2021).

[27] Almalki YE Qayyum A Irfan M Haider N Glowacz A Alshehri FM. A novel method for COVID-19 diagnosis using artificial intelligence in chest X-ray images. Healthcare. (2021) 9:522. 10.3390/healthcare9050522 33946809 PMC8145061

[28] Glowacz A. Thermographic fault diagnosis of ventilation in BLDC motors. Sensors. (2021) 21:7245. 10.3390/s21217245 34770550 PMC8587833

[29] Naseem R Shah MA Ahmad A Shaukat Z Irfan M Muhammad F. Empirical assessment of machine learning techniques for software requirements risk prediction. Electronics. (2021) 10:168. 10.3390/electronics1002016831450113

[30] Hussain A Tariq S Draz U Ali T Irfan M Rahman S. Waste management and prediction of air pollutants using IoT and machine learning approach. Energies. (2020) 13:3930. 10.3390/en13153930

[31] Glowacz A. Thermographic fault diagnosis of shaft of BLDC motor. Sensors. (2022) 22:8537. 10.3390/s22218537 36366235 PMC9656265

[32] Smith JW Everhart JE Dickson WC Knowler WC Johannes RS. Using the ADAP learning algorithm to forecast the onset of diabetes mellitus. In: Proceedings of the Symposium on Computer Applications and Medical Care. IEEE Computer Society Press (1988). pp. 261–5.

[33] Mujumdar A Vaidehi V. Diabetes prediction using machine learning algorithms. Procedia Comput Sci. (2019) 165:292–9. 10.1016/j.procs.2020.01.047

[34] Hayashi Y Himeno T Shibata Y Hirai N Asada-Yamada Y Sasajima S. Simplified electrophysiological approach combining a point-of-care nerve conduction device and an electrocardiogram produces an accurate diagnosis of diabetic polyneuropathy. J Diabetes Investig. (2024) 15:736–42. 10.1111/jdi.14174 38421109 PMC11143421

[35] Ke J Li K Cao B. A Nomogram for predicting vision-threatening diabetic retinopathy among mild diabetic retinopathy patients: a case–control and prospective study of type 2 diabetes. Diabetes Metab Syndr Obes. (2023) 16:275–83. 10.2147/DMSO.S394607 36760600 PMC9888403

[36] Chang Z Du Z Zhang F Huang F Chen J Li W. Landslide susceptibility prediction based on remote sensing images and gis: comparisons of supervised and unsupervised machine learning models. Remote Sens. (2020) 12:502. 10.3390/rs12030502

[37] Jakka A Rani JV. Performance evaluation of machine learning models for diabetes prediction. Int J Innov Technol Explor Eng. (2019) 8. 10.35940/ijitee.K2155.0981119

[38] Saeedi P Petersohn I Salpea P Malanda B Karuranga S Unwin N. Global and regional diabetes prevalence estimates for 2019 and projections for 2030 and 2045: results from the International Diabetes Federation Diabetes Atlas. Diabetes Res Clin Pract. (2019) 157:107843. 10.1016/j.diabres.2019.107843 31518657

[39] Tigga NP Garg S. Prediction of type 2 diabetes using machine learning classification methods. Proced Comput Sci. (2020) 167:706–16. 10.1016/j.procs.2020.03.336

